# Supplementary material for: β-Variational autoencoders and transformers for reduced-order modelling of fluid flows
Source: Nat Commun. 2024 Feb 14;15:1361. doi: 10.1038/s41467-024-45578-4 (PMC10866995; doi:10.1038/s41467-024-45578-4)
Supplement: Supplementary file 1 — Supplementary Information [file 41467_2024_45578_MOESM1_ESM.pdf]

Supplementary information.
β-Variational autoencoders and transformers for reduced-order modelling of fluid flows

Alberto Solera-Rico[1,2], Carlos Sanmiguel Vila[1,2], M. Á. Gómez[2], Yuning Wang[3], Abdulrahman Almashjary[4], Scott T. M. Dawson[4], Ricardo Vinuesa[3]

1: Aerospace Engineering Research Group, Universidad Carlos III de Madrid, Leganés, Spain
2: Subdirectorate General of Terrestrial Systems,
Spanish National Institute for Aerospace Technology (INTA), San Martín de la Vega, Spain
3: FLOW, Engineering Mechanics, KTH Royal Institute of Technology, SE-100 44 Stockholm, Sweden
4: Mechanical, Materials, and Aerospace Engineering Department,
Illinois Institute of Technology, Chicago, IL 60616

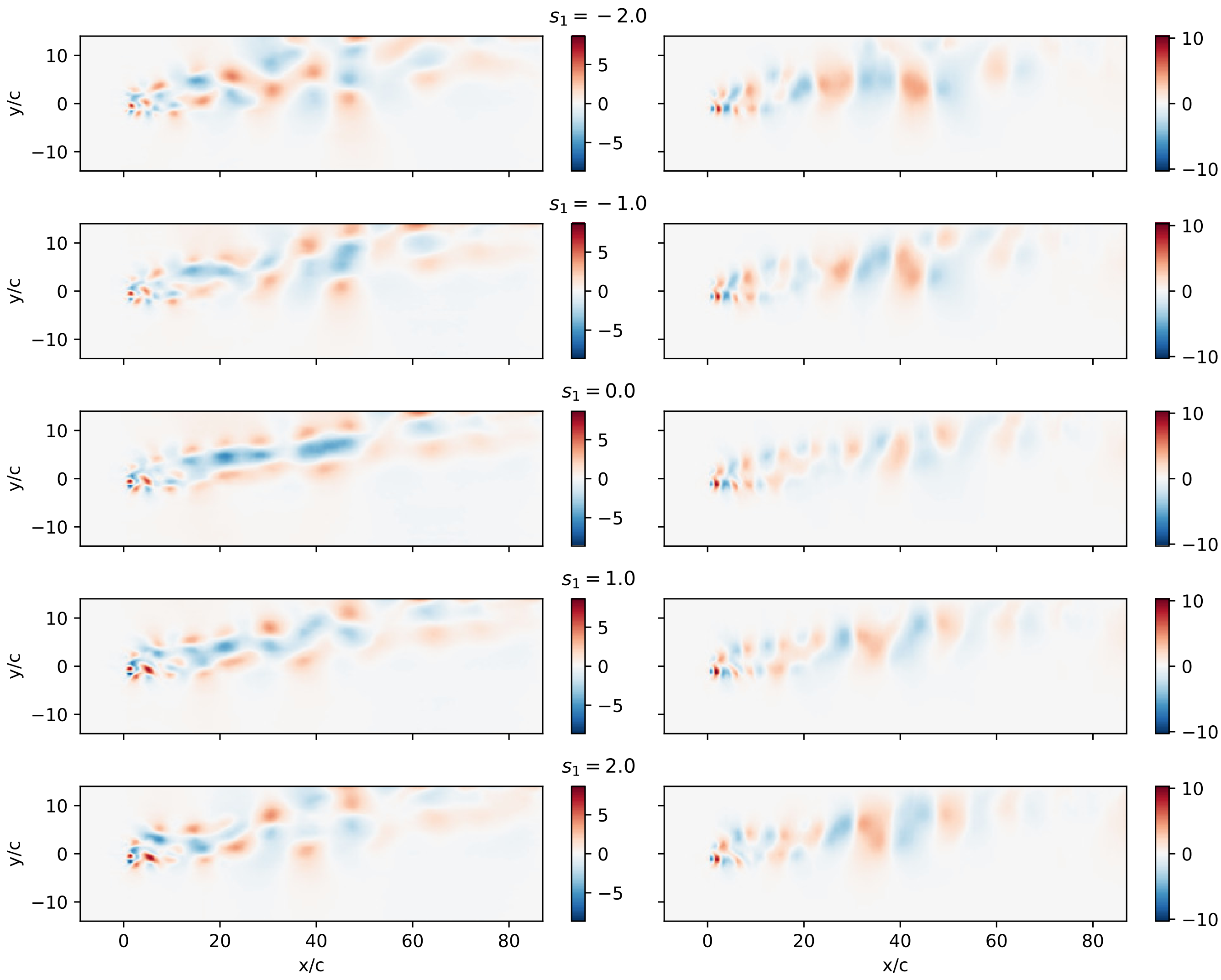

Supplementary Figure 1. **Non-linear β-VAE latent-space.** Visualisation of the first β-VAE mode with different latent input values. The first column contains the streamwise-velocity component modes and the second one the crosswise-velocity component. Case with $Re = 100$, $\alpha = 80°$
